# Global cultural evolutionary model of humpback whale song

**DOI:** 10.1098/rstb.2020.0242

**Published:** 2021-10-25

**Authors:** Lies Zandberg, Robert F. Lachlan, Luca Lamoni, Ellen C. Garland

**Affiliations:** ^1^ Department of Psychology, Royal Holloway University of London, Egham Hill, Egham TW0 0EX, UK; ^2^ Centre for Social Learning and Cognitive Evolution, and Sea Mammal Research Unit, Scottish Oceans Institute, School of Biology, University of St Andrews, St Andrews, Fife KY16 8LB, UK

**Keywords:** vocal learning, individual-based simulations, song, cultural evolution, cultural transmission

## Abstract

Humpback whale song is an extraordinary example of vocal cultural behaviour. In northern populations, the complex songs show long-lasting traditions that slowly evolve, while in the South Pacific, periodic revolutions occur when songs are adopted from neighbouring populations and rapidly spread. In this species, vocal learning cannot be studied in the laboratory, learning is instead inferred from the songs' complexity and patterns of transmission. Here, we used individual-based cultural evolutionary simulations of the entire Southern and Northern Hemisphere humpback whale populations to formalize this process of inference. We modelled processes of song mutation and patterns of contact among populations and compared our model with patterns of song theme sharing measured in South Pacific populations. Low levels of mutation in combination with rare population interactions were sufficient to closely fit the pattern of diversity in the South Pacific, including the distinctive pattern of west-to-east revolutions. Interestingly, the same learning parameters that gave rise to revolutions in the Southern Hemisphere simulations gave rise to evolutionary patterns of cultural evolution in the Northern Hemisphere populations. Our study demonstrates how cultural evolutionary approaches can be used to make inferences about the learning processes underlying cultural transmission and how they might generate emergent population-level processes.

This article is part of the theme issue ‘Vocal learning in animals and humans’.

## 1. Introduction

Social learning underpins a wide variety of behaviours in many species of animal. Social learning (from the observation of, or interaction with, others) can lead to innovations spreading through a population (e.g. [1–4]) or could, on longer timescales, potentially lead to the emergence of local cultures (e.g. [5–9]). One example of social learning, vocal production learning, has been extensively studied in birds and to some extent mammals. Birdsong is the most studied example of vocal learning, with all species of songbird that have been tested experimentally having been shown to socially learn the (components of) songs that function in resource defence and breeding [10]. But only a small proportion of species have been studied experimentally in laboratory conditions. Instead, evidence for the ubiquity of vocal learning within the songbirds has been augmented by field studies that infer vocal learning from patterns of geographical variation in song, or changes in the frequency of song types over time. Such studies of cultural transmission and evolution have been carried out in numerous species [11] (e.g. corn buntings, *Emberiza* (*Miliaria*) *calandra* [12]; white-crowned sparrows, *Zonotrichia leucophrys* [13]; village indigobirds, *Vidua chalybeata* [14]; song sparrows, *Melospiza melodia* [15]; indigo buntings, *Passerina cyanea* [16,17]; savannah sparrows, *Passerculus sandwichiensis* [18]; chaffinches, *Fringilla coelebs* [19] and swamp sparrows, *Melospiza georgiana* [20]). Like in songbirds, captive studies of smaller cetacean species have demonstrated that they are capable of vocal production learning, where as a result of experience with signals of other individuals, an individual modifies its own signal [21]. But just as with songbirds, not all cetacean species have been or could be studied in the laboratory or are amenable to field experiments. Included in this category is a species whose complex [22], evolving [23,24] song displays represent an extraordinary example of vocal cultural behaviour, the humpback whale (*Megaptera novaeangliae*) [25].

Male humpback whales sing, at any timepoint, a single long, stereotyped, complex and hierarchically structured song [22–24]. Although the exact details are still debated (see [26]), song in humpback whales is thought to function in sexual selection. The song is arranged in a nested hierarchy: on the most fundamental level is the ‘unit’, representing an individual sound; a few units sung in a stereotyped sequence constitute a ‘phrase’; phrases are repeated one to many times to make a ‘theme’; and finally, a sequence of different themes makes up a song [22,27]. Within a population, at any point in time, most males will sing the same song (known as a ‘song type’) [28,29]. At the same time, however, the song is also constantly evolving [7,29,30]; males incorporate changes into the population song in their own display to maintain the observed conformity. Slow, progressive song evolution is a key feature of all humpback whale populations worldwide. What processes (e.g. production errors, innovations), learning biases or individual(s) may be driving this evolutionary change, and how this may relate to fitness, female choice and reproductive success, remains elusive [25].

Another key feature of humpback whale song is at the scale of oceans: within oceans, populations sing similar songs but the degree of similarity depends on the geographical distance and also appears to depend on the extent of interchange among populations [31–33]. Humpback whales spend the summer months feeding in high latitudes before undertaking one of the longest migrations of any mammal to their low latitude winter breeding and calving grounds, where they aggregate around islands and banks [34–38]. They show strong maternally directed site fidelity to breeding and feeding grounds, with occasional movement among locations [39–42]. Song sharing among populations is suggested to occur as a result of three mechanisms [31], which have been demonstrated to varying degrees around the world [39,42–49]. Song sharing between populations can occur through males visiting more than one wintering ground in consecutive years, by males visiting more than one wintering ground within a breeding season, and finally through song sharing on shared feeding grounds and/or on shared or partially shared migratory routes [31]. This can result in a common (single), ocean-wide song type that has varying degrees of similarity (based on ‘matching’ themes/phrase types), as epitomized by studies in the North Pacific (see [33]).

However, in 1996 and 1997, a cultural phenomenon was recorded for the first time that represented a very different pattern of variation. Song from the west Australian humpback whale population, located in the Indian Ocean, appeared in the east Australian population, in the South Pacific, and rapidly replaced the very different existing song [50]. This process, in which the song in a population is rapidly replaced by a completely novel song, was termed a ‘song revolution’, to distinguish it from the much slower process of song evolution [50]. The new song first appeared in low numbers and then increased in frequency until the old song was completely gone; a process that took 2 years [50]. The authors hypothesized that the movement of a small number of whales from the west Australian population to the east Australian population may have initiated the cultural transmission of the song between the oceans [50]. Further work demonstrated the expansion of this phenomenon where multiple song types and thus revolutions have been horizontally transmitted east from the east Australian population across the populations in the South Pacific, in a series of cultural waves spanning a decade [7,43,51,52]. Multiple song types are thus present in the South Pacific Ocean at any point in time in contrast to the single ocean song type characteristic of the Northern Hemisphere oceans. This pattern of not only population-wide concerted song evolution but also revolution in humpback whales is unique among non-human animals [25].

**Figure 1.**
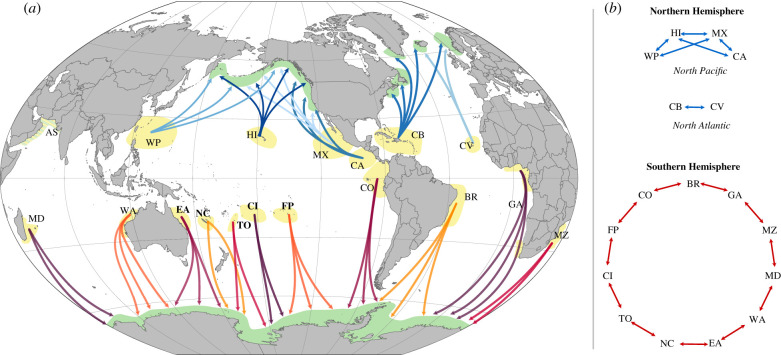
(*a*) World map showing suggested breeding and feeding areas of humpback whales [56–58]. Arrows indicate to which general feeding areas whales from the different breeding stocks migrate, with blue shades representing the Northern Hemisphere populations and red shades indicating the Southern Hemisphere populations (arrows are not intended to indicate exact migratory routes). It is highly unlikely that whales from the Northern and Southern Hemisphere populations come into contact with each other, owing to the difference in seasons between the two Hemispheres (winter = low latitude breeding areas, summer = high latitude breeding areas). Northern Hemisphere breeding populations: WP, West Pacific; HI, Hawaii; MX, Mexico; CA, Central America; CB, Caribbean; CV, Cape Verde; AS, Arabian Sea (non-migratory population, not included in model). Southern Hemisphere breeding populations: BR, Brazil; GA, Gabon and West South Africa; MZ, Mozambique; MD, Madagascar, La Réunion; WA, west Australia; EA, east Australia; NC, New Caledonia; TO, Tonga; CrI, Cook Islands (possibly migratory); FP, French Polynesia; CO, Colombia, Costa Rica, Panama, Ecuador. Populations from which the empirical data were collected are indicated in bold. (*b*) Interaction patterns as implemented in the model. Arrows represent the presence of interactions. In the model, the Southern Hemisphere populations were evenly spaced and connected only with the neighbouring populations on either side. The Northern Hemisphere populations in the model were allowed to interact with, depending on the population, all or almost all other populations within their respective oceans.

To date, the only event that appears to trigger a song revolution is the appearance of a new song type in a population that can be traced to come from another [7,50]. We hypothesize that novel songs are rapidly and preferentially learned [50,53]. This might result from a sexually selected drive for novelty [30,50]. However, if this is the case, that raises a problem: what constrains songs within populations from diverging from each other [54]? It is not clear whether a neutral process of cultural mutation and transmission could also give rise to revolutionary dynamics. We speculate that song revolutions occur throughout the Southern Hemisphere [25], with recent hints of directional transmission also emerging around Africa [55]. This may be facilitated by geographical structure: the large circumpolar feeding grounds lead to low rates of contact between neighbouring populations, while providing no landmasses to impede movement at high latitude. Moreover, the west-to-east direction of the song revolutions observed in the South Pacific is hypothesized to occur owing to the relative differences in population sizes. Novel song types appear to spread from large to small populations [7,52]. On the other hand, the fact that revolutionary dynamics are empirically absent in Northern Hemisphere populations (primarily located in the North Pacific and North Atlantic) may be due to differences in learning biases, or alternatively could be due to differences in geography that affect the patterns of population interactions. The Northern Hemisphere populations are constrained by continents on each side of the oceans, which prevents interaction between Atlantic and Pacific meta-populations, while at the same time encourages multiple populations to interact within an ocean by funnelling populations into comparatively small areas (figure 1*a*) [25]. Experimentally testing such hypotheses is unfeasible; humpback whales cannot be kept in captivity, as they are 14 m long, weigh 30 tonnes and undertake long migrations across half the globe. They can also be less than amenable to field experiments, notwithstanding the ethical considerations surrounding attempting to modify a vocal cultural display that may spread at the Hemispherical scale [59]. A viable way forward is to use cultural evolutionary models in combination with empirical data to infer which processes could underlie these broad-scale patterns of cultural transmission. Recent work using spatially explicit agent-based models of fine-scale song evolution, taking into account the spatial movements of individual whales, suggested that production errors (rate = 0.01%) led to the pattern of gradual song evolution observed in the wild [60]. To produce a song revolution that mirrored the empirical data from the west–east Australian song revolution, a song memory was included [61]. These fine-scale models on song transmission within and between the west Australia and east Australia populations explored learning processes and biases to understand, at the level of the individual, what factors may facilitate song evolution and revolution.

Here, we modelled humpback whale song transmission, not at a population scale, but at the global Hemisphere scale, to understand what broad-scale processes may initiate, stop, and spread song evolutions and revolutions. Using individual-based simulations (based on [20]), we explored how past population trajectories [56] in combination with connectivity and geographical location may interact to create conditions that promote cultural dynamics. As any model has necessary simplification, we compared our model with data from published South Pacific humpback whale research (e.g. [7,51,62]), using approximate Bayesian computation (ABC) to find parameter values that were statistically most consistent with the empirical data. We also compared our model more informally with patterns of song sharing in other oceans [22,29,33,55,63,64]. By using models to explore likely scenarios, targeted future field studies can be designed to empirically test hypotheses.

## 2. Methods

### (a) Empirical song data

Empirical song data was drawn from published studies of humpback whale song transmission in the South Pacific Ocean. Detailed descriptions of field locations, data collection methods, song transcription and quantitative analyses can be found in [7,51,62]. Briefly, 211 singers were recorded singing 798 songs from 1998 to 2008 across the western and central South Pacific populations [51]. Units were transcribed and then grouped in stereotyped themes; ‘matched’ themes were given the same label, and a sequence of themes made up each song. Songs with similar themes were grouped into song types [7]. Here, the presence of themes per singer was collated regardless of whether those themes were common or not. Song length ranged between 2 and 11 themes with 6.84 ± 1.73 (mean ± s.d.) themes per song.

### (b) Model 1—cultural evolutionary model of song learning in the Southern Hemisphere

We constructed an individual-based simulation model of song learning and cultural evolution. For a summary overview of all models see table 1.

**Table 1.** Summary overview of the different models.

model		description
1	Southern Hemisphere (SH)	Full model of song learning and cultural evolution in the 11 Southern Hemisphere (SH) populations with the associated population size estimates. Using ABC we obtained the posterior distribution of the model parameters by comparing the model outcomes with the empirical data. All other models use song learning parameters sampled from this posterior distribution.
1a–c	population size and direction of revolutions	Exploring the effect of relative population sizes in the SH on the direction of revolutions by varying the different population size estimates:
	(*a*) rerun of SH: empirical population sizes	Owing to stochasticity, rerunning the simulation with the same parameters does not always give the same results. We used the original population sizes *N*_*i*,min_, *N_i_*_,2015_ and *N_i,*K*_*.
	(*b*) equal population sizes	To test the hypothesis that relative differences in population size determine the direction of the revolutions, we set the *N*_*i*,min_, *N_i_*_,2015_ and *N_i,*K*_* for all populations to the same values (*N*_*i*,min_ = 100, *N_i_*_,2015_ = 1000 and *N_i,*K*_* = 5000)
	(*c*) bottleneck: *N*_min_ empirical size, *N_i_*_,2015_ and *N_i,K_* equal	To explore whether the bottleneck in population sizes affected the direction of the revolutions, we set the *N_i_*_,2015_ and *N_i,*K*_* to equal values, and kept the *N*_*i*,min_ the same as the empirical *N*_*i*,min_.
2	projecting cultural evolution into the future	To explore what would happen to the patterns of song evolution and revolution we reran Model 1, but in this case let it run for a total of 500 years, while allowing the populations to reach carrying capacity.
3	Northern Hemisphere	To test whether the SH learning parameters would also lead to patterns of song transmission as found in the Northern Hemisphere (NH), we reran Model 1 using the NH populations, their population sizes and patterns of interaction.

#### (i) Population structure

We simulated all humpback whale populations in the Southern Hemisphere: 11 populations in a circular chain. Each population had a population to its left and a population to its right (figure 1*b*); all distances between populations were the same for ease of modelling. We based the size of the populations on [56], with the exception of Oceania, which we divided into four smaller populations (New Caledonia, Tonga, American Samoa/Niue/Cook Islands and French Polynesia) to be able to compare these with the empirical data. All population size estimates were divided by 2 to represent only the males in these populations. We simulated cultural evolution over a period of 50 years, starting at a whaling-induced population ‘bottleneck’ in 1960. For each population, *i*, we estimated three different population sizes: *N_i_*,_min_ (estimated minimum abundance during the bottleneck), *N_i_*_,2015_ (estimated abundance in 2015) and *N_i,_*_K_ (estimated carrying capacity), based on estimates in [56]. Each simulation ran for 50 years during which populations were allowed to grow from *N_i,_*_min_ to their *N_i_*_,2015_ with a population-dependent logistic growth rate estimated from the three population sizes. See electronic supplementary material, table S1 for all population size estimates used in the models (electronic supplementary material, S1).

#### (ii) Songs and song memory

In the simulation model, songs were characterized by a string of 11 units representing the themes in a song. Some theme locations in the array could be left ‘empty’ representing the absence of a theme, to allow for differences in song lengths: ranging between 2 and 11 themes per song. Each individual whale in the model at any timepoint (epoch) only sang one song: its ‘current song’. Whenever an individual learned a new song, this song was stored in its song memory. The length of the song memory in epochs, *L*_sm_, was drawn from the priors. When learning a new song, the oldest song in the memory was replaced by the new song. Only current songs were sung at any timepoint, with the memory only serving as a repository of old songs against which tutor songs were compared (see §2b(iv), ‘Song selection and song learning’). At the start of each simulation, all individuals within a given population started with the same song consisting of seven different themes, which differed for each population. As our models measure theme sharing (see §2b(v) ‘Summary statistics’), a revolution was deemed to occur when the within-population theme sharing with the previous year was very low. This suggested that within a year the old themes (and thus the old song type) were rapidly replaced by new themes.

#### (iii) Learning epochs

Humpback whales predominantly sing during the breeding season, although some song has also been heard during migration and on the feeding grounds [48,65]. Here, during migration and on the feeding grounds, rare interactions between individuals of different populations may provide opportunities for exposure to song from neighbouring populations [44,45]. To mimic these patterns of singing behaviour and interactions in our model, each year individuals went through 10 song learning events (learning epochs). Of these 10 epochs per year, nine epochs corresponded with the breeding season, during which individuals only interacted with individuals from their own population as potential tutors. Each year one of the learning epochs corresponded with the migratory/feeding season during potential interactions with other populations, and tutors could be selected from a neighbouring population.

#### (iv) Song selection and song learning

During a learning epoch a focal individual randomly selected *N*_t_ tutors. In the nine breeding season learning epochs, these tutors were randomly drawn from the focal's own population. In the single migratory/feeding season epoch each tutor was drawn randomly with a probability *P*_n_ for each tutor to come from a neighbouring population. Individuals only selected novel songs for learning (songs that were not already present in their memory): each of the songs sung by the selected tutors was compared with each of the songs in the memory of the focal individual. For each of these comparisons, if a given tutor's song contained one or more themes that were not present in a given focal song, it was considered a different song. Only when all songs in the focal individual's song memory were different from the tutor's song—and it was considered a novel song—could it be learned. If one or more novel songs were sampled by a whale during a learning epoch, one was selected at random and copied by the focal individual into its song memory. Copying of songs did not always happen faithfully: deletions, insertions and theme substitutions occurred. Firstly, for each theme in the chosen song there was a probability *P*_learn_ for each theme that it was learned (and not deleted from the song). This probability was dependent on the number of times *k* that the theme was present in the tutor songs the focal individual heard during that epoch, and a parameter for deleting themes, *P*_d_: $P_{\mathrm{learn}}=1/(1+0.5^{\left(k+P_{d}\right)})$. When a theme was deleted, this location in the song array was left empty. Secondly, for each empty location in the song array, a new theme could be invented and inserted with a probability calculated as: $P_{\mathrm{insert}}=e^{-P_{d}P_{i}}$, where *P*_i_ was drawn from the priors. Lastly, theme substitutions occurred with a substitution rate *μ*, in which a theme was substituted by a new theme. In cases where all tutor songs were already present in the focal individual's song memory, no new song was learned. Instead, only new theme substitutions were introduced in the old song, with substitution rate *μ*, similar to newly learned songs. These changes—insertions, deletions and substitutions—broadly mirror humpback song evolution processes. To prevent an effect of the order in which the populations go through their learning epochs, each individual's current song was only replaced by a newly learned song after all individuals had gone through the song selection and song learning phases in that epoch.

#### (v) Summary statistics

To compare the empirical data with the simulated data we measured seven summary statistics, averaged over all populations and the last 11 simulated years. We compared these with the same statistics measured on the empirical data. The sample sizes measured for each population and year followed the sample sizes in the 11 years of the empirical data: each sample represented 1 year of one particular population. All summary statistics were calculated on the basis of the song that each particular individual in that population sample sang in that particular epoch (not on the basis of its memory). Summary statistics consisted of: (1) singleton themes: average number of theme types that were sung by only one individual in a sample population (sp); (2) the unweighted average of the average song length in a sp; (3) the within-population standard deviation of the song lengths. Statistics (4)–(7) were derived from theme sharing between different populations and/or timepoints. Theme sharing was calculated following formula (2.1), where ns*_A_* is the sample size of the sp, and ns*_B_* the sample size for the population it is compared with. *t_A,j_* indicates the themes for individual *j* in the sp, and *t_B,k_* the themes of individual *k* in the population it is compared with:

2.1 $${\mathrm{TS}}_{A,B}=\left(\overset{{\mathrm{ns}}_{A}}{\underset{j}{\sum }}\left(\overset{{\mathrm{ns}}_{B}}{\underset{k}{\sum }}|t_{A,j}\cap t_{B,k}|/|t_{A,j}|\right){\mathrm{ns}}_{B}^{-1}\right){\mathrm{ns}}_{A}^{-1}.$$

Summary statistic (4) was the average of theme sharing within the sp (TS_FC_): calculated from formula (2.1), with population *B* = population *A*, but excluding cases where *j* = *k*. Summary statistic (5) is the average of the theme turnover within the sample population, calculated as the change in average theme sharing within the sp between the current year and the previous year: ${\mathrm{TS}}_{\mathrm{sp},\mathrm{sp}}-{\mathrm{TS}}_{\mathrm{sp},\mathrm{sp}(\mathrm{year}-1)}$. Summary statistic (6) is the average of the absolute difference in theme sharing between the sample population and both neighbouring populations (western population WP and eastern population EP), in the previous year: ${\mathrm{TS}}_{\mathrm{sp},\mathrm{sp}(\mathrm{year}-1)}-{\mathrm{TS}}_{\mathrm{sp},\mathrm{ep}(\mathrm{year}-1)}$. Finally, summary statistic (7) is the average of the maximum theme sharing between the sp and both neighbouring populations in the previous year: Max TS_WP_,TS_EP_. We square root transformed summary statistics (1), (3), (5) and (7). We then normalized each summary statistic by dividing it by the standard deviation of this statistic in a set of 3000 simulations sampling from the priors.

#### (vi) Approximate Bayesian computation

We used ABC to obtain the posterior distribution of the model parameters by comparing the summary statistics of the empirical data with the summary statistics of the simulated data [20,66]. Simulations were carried out with parameter values drawn from the prior distributions (see below). For each simulation, we then calculated the Euclidian distance across the normalized summary statistics of the simulated data and the empirical data. Parameter values were accepted when the summary statistic values of the simulated data were within threshold *ε* from the empirical summary statistic values. By using a population Monte-Carlo approach to the ABC model (PMC-ABC; [66]) we were able to reduce the number of simulations needed to obtain a sufficient number of simulations within the final threshold *ε*. PMC-ABC achieves this by carrying out subsequent sets of simulations in which the threshold *ε* is stepwise decreased. We used the following values for *ε*: {6, 5.5, 5, 4.5, 4, 3.5, 3, 2.75, 2.5}. We ran each set until 1000 accepted parameter sets were produced.

#### (vii) Prior distributions

We set the prior distributions for *N*_t_, *P*_n_, *μ* and *L*_sm_ as log-uniform distributions with limits *N*_t_ {1, 20}; *P*_n_ {0.0000001, 0.25}; *μ* {1 × 10–10, 0.001}; *L*_sm_ {1, 100}. *P_*d*_* and *P_*i*_* were given a uniform distribution with limits *P_*d*_* {1, 20}; *P_*i*_* {0.2, 6}.

#### (viii) Validation of ABC

To validate that our ABC design was producing unbiased parameter estimates we carried out a leave-one-out cross-validation analysis. We used the 73 600 samples from the adjusted priors in the last round of the PMC method, and for each one of these, estimated its parameter values using the remaining samples. We describe these results in electronic supplementary material, S2.

### (c) Model 1a–c—population size and direction of revolutions

To test the hypothesis that relative differences in the population sizes underlie the west-to-east direction of the revolutions, we further explored the effects of population size, and population growth on the direction of revolution transmission. We ran 1200 simulations for each of three different scenarios (a–c: see also table 1) using song learning parameters sampled from the posterior distribution of the Southern Hemisphere in Model 1. (*a*) We reran the simulations for the Southern Hemisphere, because, owing to stochasticity, we did not expect the same parameter settings to always generate the same theme sharing patterns. For this simulation, we used the original population sizes *N*_*i*,min_, *N_i_*_,2015_ and *N_i,*K*_* as described in Model 1. (*b*) To explore the effect of relative differences in population sizes on the direction of the revolutions we ran the same model but with all population sizes set to equal values, to see whether the pattern of eastward revolutions would disappear. All populations started with *N*_*i*,min_ = 100, and grew to *N_i_*_,2015_ = 1000 and *N_i,*K*_* = 5000. (*c*) Finally, we also explored the role of the population size bottleneck on the direction of the revolutions. For this, we reran the simulations for the Southern Hemisphere but only fixed *N_i_*_,2015_ for all populations to 1000 individuals, and *N_i,*K*_* to 5000 individuals, while *N*_*i*,min_ was kept at the empirical values. For each model, we selected the simulations with *ε* < 3, and calculated the proportion of revolutions that ran from east to west (opposite to the direction found in the empirical data).

### (d) Model 2—projecting cultural evolution into the future

In the second model, we explored what would happen to the patterns of song evolution and revolution in the future, if population sizes were to increase and approach carrying capacity. In this model, we ran the simulations for 500 years (starting during the 1960 bottleneck, and projecting *ca* 450 years into the future), and allowed populations to grow to their carrying capacity. For this model, we sampled with replacement 1000 parameter settings from the posterior distribution of Model 1—i.e. settings that generated summary statistics that were a close fit to the empirical data. For each simulation, we calculated average theme sharing within the focal population, and between the focal population and each of the neighbouring populations in the previous year.

### (f) Model 3*—*Northern Hemisphere

In the third model, we compared the differences in patterns of theme sharing between the Northern Hemisphere and the Southern Hemisphere humpback whale populations. For this we ran 1000 simulations for both the Northern and the Southern Hemisphere populations using song learning parameters sampled from the posterior distribution of the Southern Hemisphere in Model 1. We reran the simulations for the Southern Hemisphere, because, owing to stochasticity, we did not expect the same parameter settings to always generate the same theme sharing patterns. For the Northern Hemisphere simulations, we used the exact same model as for the Southern Hemisphere (see Model 1), but only changed the number of populations, population size estimates and the pattern of interactions between the populations to match those observed in the Northern Hemisphere (figure 1*b*; electronic supplementary material, table S1). We used current population size estimates for the different populations in the Northern Pacific and Atlantic oceans [57], and, since these are not as readily available as for the Southern Hemisphere, we roughly estimated bottleneck and carrying capacity population sizes. Interaction patterns between populations on the feeding grounds were based on [57,67]: both populations in the Atlantic Ocean and most populations the Pacific Ocean were allowed to interact with each other, but there were no interactions between Atlantic and Pacific populations. For the interaction rate between the populations, we used the estimate obtained from Model 1. While there is no reason to expect that this parameter value is the same in the Northern Hemisphere as the Southern Hemisphere, we chose to use this value out of conservatism. For each simulation, we calculated within-population theme sharing and turnover. We also calculated average theme sharing between the focal population and each of the neighbouring populations in the previous year, and the difference in theme sharing with the eastern and western neighbouring populations (Diff TS_WP_ − TS_EP_: ${\mathrm{TS}}_{\mathrm{sp},\mathrm{sp}(\mathrm{year}-1)}-{\mathrm{TS}}_{\mathrm{sp},\mathrm{ep}(\mathrm{year}-1)}$).

## 3. Results

### (a) Model 1*—*Southern Hemisphere

**Figure 2.**
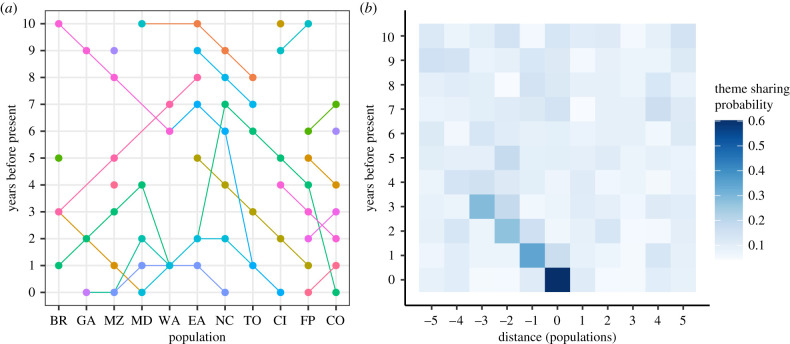
Southern Hemisphere model theme sharing. (*a*) Theme trajectories in the different populations and over the different years, with years before. Years 0–10 represent the last year in the simulation (or the current year), and the 10 years before that. Different colours indicate different themes. (*b*) Theme sharing profile with the focal population = 0, and the populations west (negative values) and east (positive values) of the focal population. The *y*-axis represents the years before the last simulated year (current = 0 years before). Colour intensity indicates the average probability of theme sharing between the focal population in the current year and the population and year indicated by the *x*- and *y*-axes.

With relatively simple learning rules we were able to create a model with signatures of both evolution and revolution of songs. Within-year, within-population theme sharing was high (TS_FC_ median: 0.67, 95% credible interval (CrI): 0.53–0.82), whereas within-population theme sharing with the previous year was low (TS_FP_ median: 0.15, CrI: 0.03–0.37), suggesting a high rate of turnover of themes present in the population. Theme sharing with neighbouring populations in the previous year was higher than within-population sharing in the previous year, with maximum theme sharing (regardless of which neighbour) of 0.32 (Max TS_WP_,TS_EP_ CrI: 0.24–0.45), suggesting revolutionary changes. By calculating the difference in theme sharing with the focal population between the eastern and western neighbours, we found that the majority of the revolutions ran from west to east (Diff TS_WP_ − TS_EP_ median: 0.21, CrI: −0.21–0.34). Out of 1000 simulation runs, 552 showed a signature of revolutionary waves running from west to east (Diff TS_WP_ − TS_EP_ ≥ 0.2), whereas only 37 simulations showed revolutionary waves running from east to west (Diff TS_WP_ − TS_EP_ ≤ −0.2). Individual themes were transmitted from population to population through the years. Themes were usually only found in a population for 1 or 2 years before they were replaced with new themes. Although the direction of the majority of theme transmissions was from west to east, resulting in the larger patterns of eastward waves of revolutions, some themes were still transmitted from east to west (figure 2*a*). Moreover, owing to these revolutions, the average theme sharing probability between the focal population and the population to the west in the previous year was high, but decreased stepwise when going a year back in time year by year and moving a population further westward relative to the focal population (figure 2*b*). This pattern of sharing faded after around 5 years, after which the songs had evolved to such an extent that the theme sharing probability with the focal population in the current year was not higher than for other populations.

From our simulations, we estimated that individual humpback whales learned their songs with a high precision (substitution rate per individual per theme per learning epoch—median: *µ* = 1.60 × 10^−7^, CrI: 4.10 × 10^−10^–8.24 × 10^−6^). Similar to the low substitution rates, insertion and deletion probabilities were also estimated to be low (median: *P_*d*_* = 6.42, CrI: 3.34–22.73); median: *P_*i*_* = 3.69, CrI: 0.56–5.84), resulting in a deletion probability of 0.006 for a theme only heard once in that learning epoch by a focal individual. We estimated that the length of an individual's memory was 54.59 epochs (CrI: 21.36–97.5). Individuals sampled 5.11 tutors to select their new songs from (CrI: 1.57–17.95). In the feeding grounds, individuals rarely encountered and learned from neighbouring populations, with a probability of *P_*n*_* = 0.001 (CrI: 8.02 × 10^−5^–2.90 × 10^−2^). The number of tutors sampled and the probability for a tutor to be selected from a neighbouring population were negatively correlated (*r* = −0.23), which is due to the fact that the more tutors an individual samples, the greater the probability that one of these comes from another population.

### (b) Model 1a–c—population size and direction of revolutions

For the three models exploring the effects of relative population sizes and growth rates, we found that the differences in size between the populations around the moment of measuring (the simulated year 2015) determined whether or not the simulations resulted in a situation with revolutions running from east to west (opposite to the direction of the patterns found in the empirical data). In Model 1a with the empirical population size estimates we found that of all the simulations with a signature of revolutionary waves (Diff TS_WP_ − TS_EP_ > |0.2|) only a very small proportion (0.05) of these revolutions ran from east to west, mirroring the patterns found in the empirical data. In Model 1b, all population sizes at *N*_*i*,min_, *N_i_*_,2015_ and *N_i,*K*_* were set to the same values for all populations, we found that a proportion of 0.54 of the revolutions waves ran from east to west. This suggests that when the relative differences in population sizes are removed, the direction of revolutions is almost equally likely to run from east to west as from west to east. We then explored the effect of the bottleneck *N*_*i*,min_ on the direction of revolutions in Model 1c, by setting the *N_i_*_,2015_ to 1000 individuals, and the *N_i,*K*_* to 5000 individuals, while keeping the *N*_*i*,min_ at the empirical values. Similar to Model 1b, the probability for revolutions to run eastward or westward was almost equal (proportion east to west = 0.54), suggesting that the bottleneck did not play a role in the direction of the revolutions in the current day.

### (c) Model 2—projecting Southern Hemisphere cultural evolution into the future

**Figure 3.**
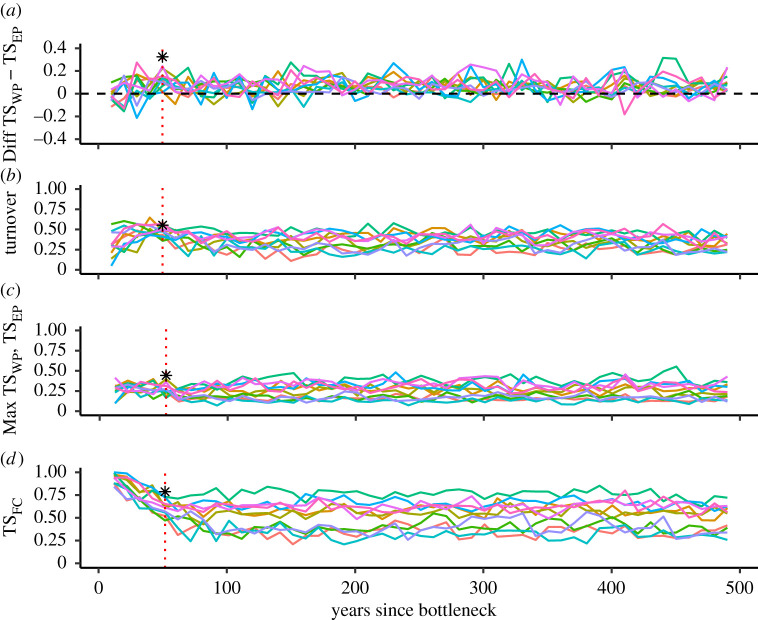
Southern Hemisphere model projecting cultural revolutions 500 years into the future; results from 10 simulation runs (each shown in a different colour). The red dotted line indicates 50 years after the bottleneck (2015) and the asterisk indicates the values for the empirical dataset. Statistics were sampled once every 10 years. (*a*) Difference in theme sharing with the focal population between the western and eastern neighbouring population in the previous year (Diff TS_WP_ − TS_EP_). Positive values indicate easterly transmission, while negative values indicate westerly transmission. (*b*) Theme turnover. (*c*) Maximum theme sharing between the focal population and the neighbouring populations in the previous year (Max TS_WP_,TS_EP_). (*d*) Within-population theme sharing within year (TS_FC_).

When we allowed models to keep running for 500 years from the bottleneck, we found that, on the basis of our model and the range of parameter settings, there was a wide range of possible future outcomes (figure 3). In a number of simulation runs, revolutionary waves could still occur, including when populations grew and approached carrying capacity. However, in most models, periods with revolutionary waves were interspersed with periods without them. When waves occurred, their direction tended to be from west to east, although some waves running from east to west were also observed (figure 3*a*). Among the predictions of our model, there were also simulations where the variation in themes within (figure 3*d*) and between populations (figure 3*c*) increased to such an extent that there were no more population-wide revolutionary waves.

### (d) Model 3—Northern Hemisphere versus Southern Hemisphere

**Figure 4.**
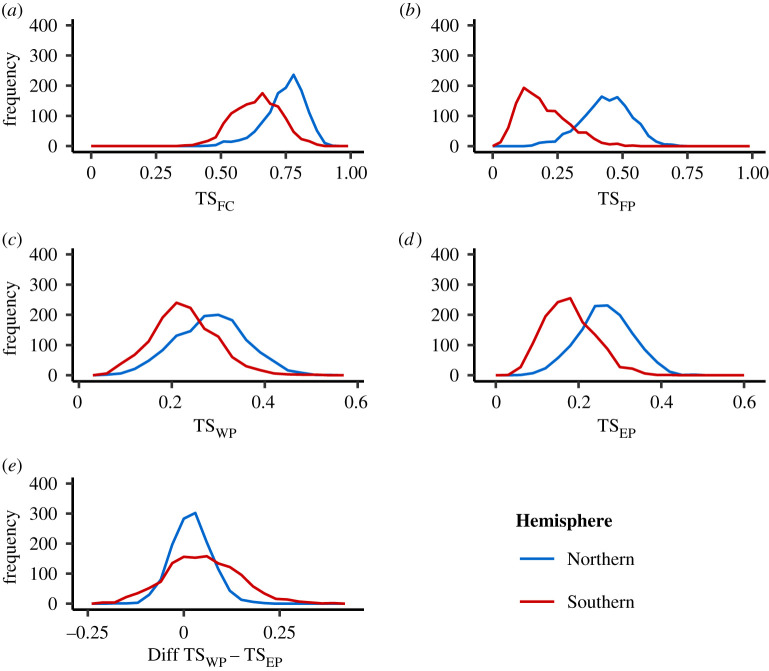
Difference in theme sharing between the Northern and Southern Hemisphere populations. Each panel shows the distribution of the different theme sharing statistics resulting from 1000 simulations. (*a*) Within-population theme sharing (TS_FC_). (*b*) Within-population theme sharing in the previous year (TS_FP_). (*c*,*d*) Theme sharing with western and eastern neighbour, respectively, in the previous year (TS_WP_ and TS_EP_). (*e*) Difference in theme sharing with the focal population between the western and eastern neighbouring population in the previous year (Diff TS_WP_ − TS_EP_). Positive values indicate higher theme sharing with a western population, while negative values indicate higher theme sharing with an eastern population.

When comparing the model outcomes for the Northern and the Southern Hemispheres we found very different patterns of theme sharing (summarized in figure 4). The Northern Hemisphere had slightly higher levels of within-population theme sharing than the Southern Hemisphere in the current year (TS_FC_: NH 0.76, SH 0.64; figure 4*a*). This difference between Hemispheres was far more pronounced in the level of within-population sharing with the previous year. The Southern Hemisphere had a much lower level of within-population sharing with the previous year (TS_FP_: NH 0.44, SH 0.17; figure 4*b*). This resulted in a higher theme turnover in the Southern Hemisphere (turnover: NH 0.30, SH 0.45), suggesting more abrupt changes in themes sung by the Southern Hemisphere populations. Moreover, theme sharing was more directional in the Southern Hemisphere than in the Northern Hemisphere. In the Northern Hemisphere, theme sharing was similar for eastern (TS_EP_) and western neighbours (TS_WP_) (TS_EP_: 0.27; TS_WP_: 0.28; figure 4*c*). In the Southern Hemisphere, however, focal populations showed a higher level of sharing with western neighbours in the previous year (TS_EP_: 0.17; TS_WP_: 0.22; figure 4*d*). This difference in sharing with the eastern and western populations (figure 4*e*), resulted in revolutionary waves in 69 of the 1000 simulations in the Southern Hemisphere (Diff TS_WP_ − TS_EP_ > |0.2|), whereas in the Northern Hemisphere, only two simulations were found showing a signature of revolutions.

## 4. Discussion

With simple song learning rules, we have been able to replicate the patterns of the cultural evolution of humpback whale song as found in the Southern Hemisphere. Here, we found that rare interactions between populations combined with a song memory and a tendency to learn novel songs was sufficient to create patterns of song evolution and revolutions. As long as learning was precise and population interactions were sufficiently rare, the tendency to learn only novel songs, not present in the song memory, led to the patterns of song conformity within populations. We found that, similar to the empirical data, the majority of our simulations resulted in revolutions running in an eastward direction, which was related to the size differences among populations. When projecting into the future we found that there was a wide range of possible future outcomes for patterns of song transmission, including both the persistence and disappearance of revolutionary waves. Moreover, using the same song learning parameters that generated revolutions in simulations of the Southern Hemisphere, we found that changing the pattern of population interactions to mimic the Northern Hemisphere led to revolutions becoming very rare, and evolutionary processes dominating.

### (a) Population interactions and direction of revolutions

As previously observed in [60,61], our models confirm that the pattern of rare interactions between populations during the non-breeding season is a key determinant as to whether or not song transmission and revolutions occur. The direction of revolutions running west to east in the Southern Hemisphere populations, which was observed both in empirical studies as well as in our simulations as an emergent property in our models, is likely to be a result of these rare interactions between populations in combination with the size differences among the populations. Novel songs are more likely to spread from larger to smaller populations (as hypothesized in [7]), resulting in eastward revolutions for the populations in the South Pacific, where population sizes decrease from west to east. This is illustrated by the results from Models 1a–c, in which the proportion of westward revolutions increased when the different populations were equal in size, in particular shortly before and during the year that was sampled. In Model 1, the population size of the focal population automatically had an effect on the number of interactions with neighbouring populations. However, in estimating interactions between neighbouring populations we took a conservative approach, by not including the effects of the population sizes of the neighbouring populations on interaction probabilities.

Our models identify the pattern of interaction between populations during the non-breeding season as being a key determinant as to whether or not revolutions occur. In these models, however, interactions are simply modelled as fixed probabilities, which is certainly an oversimplification. To move beyond this, the dynamic relationship between an individual, its conspecifics and its environment could be captured using spatially explicit agent-based models [61]. Furthermore, testing the collective effects of these individual spatial interactions can be useful to provide increasingly realistic predictions. For example, this type of model has been helpful to evaluate the effectiveness of conservation measures [68] as well as the impact of habitat degradation on individual fitness [69,70]. The modelling approach of [60] simulated both long-range humpback whale migratory movements and short-range interactions between conspecifics mediated by singing activity. An extension of this model, which includes song memory [61], is ideal to investigate the effects of population densities and individual movements on the occurrence of song revolutions between contiguous populations.

### (b) Continuation of revolutions through time

When projecting into the future we found that there was a wide range of possible future outcomes for patterns of song transmission, including both the persistence and disappearance of revolutionary waves. Revolutions could emerge, starting a particular direction of song transmission, which would persist for a number of decades before the pattern disintegrated (figure 3*a*). The presence of cultural waves in some decades and the absence in others are likely the result of stochasticity in the numbers of between-population interactions in the model. Nevertheless, our results suggest that, during a revolutionary period, song revolutions occur throughout the Southern Hemisphere, as speculated in [25]. Further, it suggests we are in a current revolutionary transmission time period in the Southern Hemisphere, as observed in empirical South Pacific song data, where multiple song types and thus revolutions have been horizontally transmitted eastward from the east Australian population across the populations in the South Pacific, in a series of cultural waves spanning a decade [7,43,51,52]. There is also a hint of directional transmission emerging around Africa [55]. Whether these cultural dynamics persist into the future will depend on the interaction with population size, carrying capacity and mixing. Overall, we hypothesize that once a revolution starts, it will continue to spread from one population to the next, and this could occur in any of the Southern Hemisphere populations. In theory, a full circumpolar transmission of a song type is possible; however, further modelling using fine-scale spatially explicit models including population density (as outlined above) may provide the key to understanding whether song transmission from small to large population sizes is possible. Regardless, a Southern Hemisphere-wide comparison of empirical song data is timely given we are currently in a time period of revolutionary dynamics.

### (c) Difference between the Northern and Southern Hemispheres

Geography matters. The Southern Hemisphere is modelled as a circumpolar ring of populations owing to the circumpolar feeding grounds around Antarctica [56], creating the potential for individuals to interact with populations on both sides (eastern and western neighbours). Two aspects of this appear critical for the emergence of revolutions: first, the lack of geographical barriers to movement between oceans, and second, the spacing out of populations so that interactions only occur between neighbouring populations. By contrast, both oceans in the Northern Hemisphere are constrained by continents on the east and west sides, so there is no contact between the two oceans. Moreover, within an ocean, land-masses funnel populations into a comparatively small area during the summer feeding season, allowing greater interaction between the multiple populations that are distant during the breeding season. Together, these factors resulted in few song revolutions emerging, high levels of song sharing among populations, and lower rates of turnover within populations. Humpback whales are vocal production learners—as a result of experience with signals (e.g. songs) of other individuals, an individual modifies its own signal [21]. The only event to date that appears to trigger a song revolution is the appearance in an ocean of a new song type that can be traced to come from another [7,50]. Here we have shown song revolutions occurred in the Southern Hemisphere models but only emerged on two (of 1000) rare occasions in the Northern Hemisphere, suggesting they are theoretically possible but highly unlikely. Simply put, without geographical barriers to whale movement, song revolutions can spread through many populations until the song evolves so much it is unrecognizable compared with the original song. Without such fluid contact, as characterized by the Northern Hemisphere, new, novel song material must evolve or be generated de novo within each population or ocean.

### (d) General points to take away about models of cultural evolution

Models can help clarify our understanding of evolutionary processes. In the case of cultural evolution, they can connect individual processes of learning to cultural processes at the level of populations. Applying techniques like ABC now allows one to fit complex computational models to empirical data, and draw statistical inferences from them too. In this case, we were able to draw conclusions about population sizes and the geographical factors that promote revolutions, as discussed above. We were also able to make inferences about the individual parameters that appear to underlie vocal learning in humpback whales. ‘All models are wrong’ [71], and in this case, refinements to our model might examine whether biases in learning (such as conformist biases) lead to a better fit between model and data. In addition, since our model only considers themes to be the same or different, we were only able to determine the upper limit to the mutation rate and not a lower limit. Future models could overcome these limitations by also modelling the acoustic structure of themes and how they are sequenced more explicitly to gain more informative predictions about mutation rates and how novelty is introduced in whale songs. Nevertheless, we believe that this model is useful in establishing that a simple mutation/drift model might be sufficient to accurately model learning in humpback whales and suggesting a range of mutation rates that are congruent with empirical patterns of diversity.

The humpback whale song transmission patterns, as found in the Southern Hemisphere, are unique among non-human animals. While song cultural evolution and local dialects are widely documented in birds [13,14,72,73], to our knowledge no other species shows a dynamic in which a whole population rapidly and concertedly replaces its song for a different version. Some bird species such as the corn bunting and the village indigobird show a similar pattern of concerted change among all males of a local song dialect [12,14]. In corn buntings, from year to year, all males concertedly make changes to the details of their local song dialect [12]. Although these song changes are evolutionary, unlike the humpback whales' song revolutions, similar processes may be driving the patterns of conformity in combination with rapid change (see [25] for a recent review). In both species, many questions remain, such as: Who or what is the source of song variation? What is the role of sexual selection in the evolution of these learning processes? and how do human-induced changes, in for instance population size, affect the patterns of cultural evolution? Taking a comparative approach in studying these questions will increase the possibilities for experimental approaches and may greatly enhance our understanding of the processes underlying cultural evolution.

To conclude, here we have found that a low level of mutations in combination with rare interactions between neighbouring populations was sufficient to closely fit the pattern of song sharing in the South Pacific, including the distinctive pattern of west-to-east revolutions. The direction of these revolutions was consistent with the relative differences in population size. Moreover, we have shown that the same learning parameters that give rise to these revolutions in the Southern Hemisphere can give rise to the evolutionary patterns of cultural evolution found in the Northern Hemisphere. These results demonstrate the potential of models of cultural evolution for making inferences about the processes underlying vocal learning and cultural transmission. Future empirical work investigating fine-scale song transmission from small to large population sizes in conjunction with extended modelling approaches including geographical distances among populations is needed to further unravel the learning processes underlying this striking pattern of cultural transmission.

## Data accessibility

The simulation code and data are available at https://github.com/lzandberg/WhaleABC.
